# Anatomical and topographical variations in the distribution of brain metastases based on primary cancer origin and molecular subtypes: a systematic review

**DOI:** 10.1093/noajnl/vdab170

**Published:** 2021-11-18

**Authors:** Tyler Cardinal, Dhiraj Pangal, Ben A Strickland, Paul Newton, Saeedeh Mahmoodifar, Jeremy Mason, David Craig, Thomas Simon, Ben Yi Tew, Min Yu, Wensha Yang, Eric Chang, Ryan P Cabeen, Jacob Ruzevick, Arthur W Toga, Josh Neman, Bodour Salhia, Gabriel Zada

**Affiliations:** 1 Department of Neurosurgery, Keck School of Medicine of University of Southern California, Los Angeles, California, USA; 2 Department of Aerospace and Mechanical Engineering, Mathematics and The Ellison Institute for Transformative Medicine of USC, Los Angeles, California, USA; 3 Department of Physics & Astronomy, University of Southern California, Los Angeles, California, USA; 4 Department of Translational Genomics, Keck School of Medicine of University of Southern California, Los Angeles, California, USA; 5 Department of Urology, Keck School of Medicine of University of Southern California, Los Angeles, California, USA; 6 Broad Stem Cell Center, University of Southern California, Los Angeles, California, USA; 7 Department of Radiation Oncology, University of Southern California, Los Angeles, California, USA; 8 USC Stevens Neuroimaging and Informatics Institute, University of Southern California, Los Angeles, California, USA

**Keywords:** brain metastases, distribution, magnetic resonance imaging, topographical variations

## Abstract

**Background:**

While it has been suspected that different primary cancers have varying predilections for metastasis in certain brain regions, recent advances in neuroimaging and spatial modeling analytics have facilitated further exploration into this field.

**Methods:**

A systematic electronic database search for studies analyzing the distribution of brain metastases (BMs) from any primary systematic cancer published between January 1990 and July 2020 was conducted using PRISMA guidelines.

**Results:**

Two authors independently reviewed 1957 abstracts, 46 of which underwent full-text analysis. A third author arbitrated both lists; 13 studies met inclusion/exclusion criteria. All were retrospective single- or multi-institution database reviews analyzing over 8227 BMs from 2599 patients with breast (8 studies), lung (7 studies), melanoma (5 studies), gastrointestinal (4 studies), renal (3 studies), and prostate (1 study) cancers. Breast, lung, and colorectal cancers tended to metastasize to more posterior/caudal topographic and vascular neuroanatomical regions, particularly the cerebellum, with notable differences based on subtype and receptor expression. HER-2-positive breast cancers were less likely to arise in the frontal lobes or subcortical region, while ER-positive and PR-positive breast metastases were less likely to arise in the occipital lobe or cerebellum. BM from lung adenocarcinoma tended to arise in the frontal lobes and squamous cell carcinoma in the cerebellum. Melanoma metastasized more to the frontal and temporal lobes.

**Conclusion:**

The observed topographical distribution of BM likely develops based on primary cancer type, molecular subtype, and genetic profile. Further studies analyzing this association and relationships to vascular distribution are merited to potentially improve patient treatment and outcomes.

**Key Points**

- Breast, lung, and colorectal cancers tended to metastasize to more posterior/caudal topographic and vascular neuroanatomical regions.
- Differences likely exist in metastasis distribution within cancers based on genetic composition and subtype.

**Importance of the Study**

Brain metastases arise in around 25% of adult cancer patients and carry a poor prognosis. While it has long been suspected that certain tumors have a predilection for certain regions of the brain, recent advances have allowed for improved analysis of this association. We conducted the first systematic literature review pertaining to topographical distributions of brain metastases based on primary cancer. Due to the numerous factors likely affecting the location of brain metastases, analyzing the results from multiple studies allows for identification of recurring trends as well as places where current literature is in disagreement. We highlight the need for further research into this field, particularly using advanced modeling techniques and larger datasets. Additionally, we emphasize the significance of understanding how the molecular and genetic makeup of primary cancer affects its predilection for a particular region of the brain.

Approximately 25% of adult cancer patients are diagnosed with brain metastases.^1^ The most common sites of origin for brain metastasis include lung (30–60%), breast (15–20%), skin (5–10%), and gastrointestinal (GI) (4–6%) cancers.^2^ The prognosis following identification of brain metastasis is poor, with an estimated overall survival between 1 and 2 months if left untreated.^3^ Current National Comprehensive Cancer Network guidelines recommend a combination of surgery, radiosurgery, whole-brain radiotherapy (WBRT), and/or systemic therapy for the treatment of brain metastasis.^4^ Treatment choice for brain metastasis is based on pathology, number and size of metastases, and location of the metastatic lesion. Surgery and/or radiosurgery has historically been more appropriate for patients with a surgically accessible or few brain metastasis while WBRT is often used for those with numerous lesions, though continued research into the effectiveness of these treatments is still being performed.^4^

It has long been hypothesized that the anatomical and topographical distribution of brain metastasis varies according to cancer subtype,^4–7^ often explained by the “seed and soil” hypothesis where the tumor cells (“seed”) metastasize to a specific area in the brain (“soil”) due to its unique microenvironment that attracts and allows the tumor to grow.^8^ More recently it has been shown that the primary cancer origin affects both the “seed” and the “soil,” influencing cellular receptor and protein expressions.^9–11^ There have been numerous emerging studies on the molecular characteristics, tumor microenvironment, and advanced neuroimaging of brain metastasis. However, no comprehensive systematic review of literature describing the tendency of a particular cancer origin to show predilection for metastasis to selected anatomical brain regions has been performed to date. Here, we examine the existing literature considering the location of brain metastasis based on primary cancer origin and analyze factors that are likely to influence their anatomical origin and distribution.

## Methods

### Literature Search

The authors conducted a systematic literature review according to Preferred Reporting Items for Systematic Reviews and Meta-Analyses (PRISMA) guidelines.^12^ MEDLINE/PubMed and Embase were queried for articles published between January 1990 and January 2021 using key terms to yield studies using magnetic resonance imaging (MRI) to investigate the location and/or distribution of brain metastasis. The keywords searched included “brain metastasis,” “brain metastases,” “location,” “distribution,” “topography,” “coordinates,” “magnetic resonance imaging,” and “MRI.” The search results were filtered for papers published after January 1990 and published in English. The references of included papers were examined, and relevant studies were identified. The PROSPERO and Cochrane databases were searched to ensure no overlapping systematic reviews had been previously published.

### Study Inclusion and Analysis

**Figure 1.**
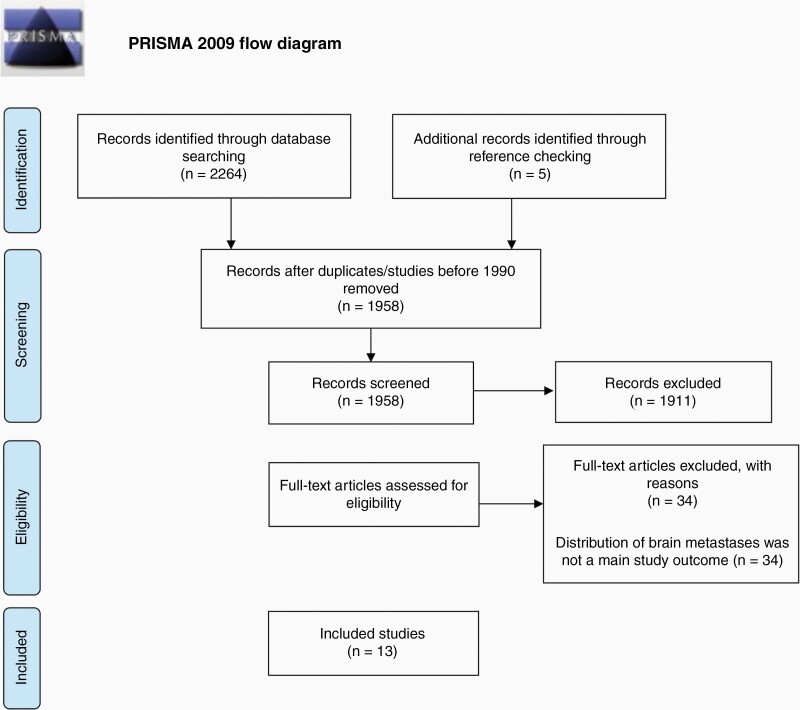
PRISMA diagram detailing the database searches, the number of abstracts screened, the full texts reviewed, and the reasons for exclusion.

After the removal of duplicate studies, 1958 articles were screened for eligibility by title and abstract. Five additional articles were subsequently identified through references of included studies. Two authors (T.C., D.P.) independently screened all article abstracts and selected potential papers for inclusion based on full-text analysis. A third author (B.A.S.) arbitrated the 2 lists via removal of duplicates and agreement with inclusion/exclusion criteria to obtain the studies included in the analysis (Figure 1). Studies were included if a primary analysis examined brain metastasis distribution and/or location from any primary cancer. Studies were excluded if they were published before 1990, not in English, not peer-reviewed, case reports/letters/commentaries, abstracts-only, cadaver/autopsy studies, or had a sample size less than 20.

**Figure 2.**
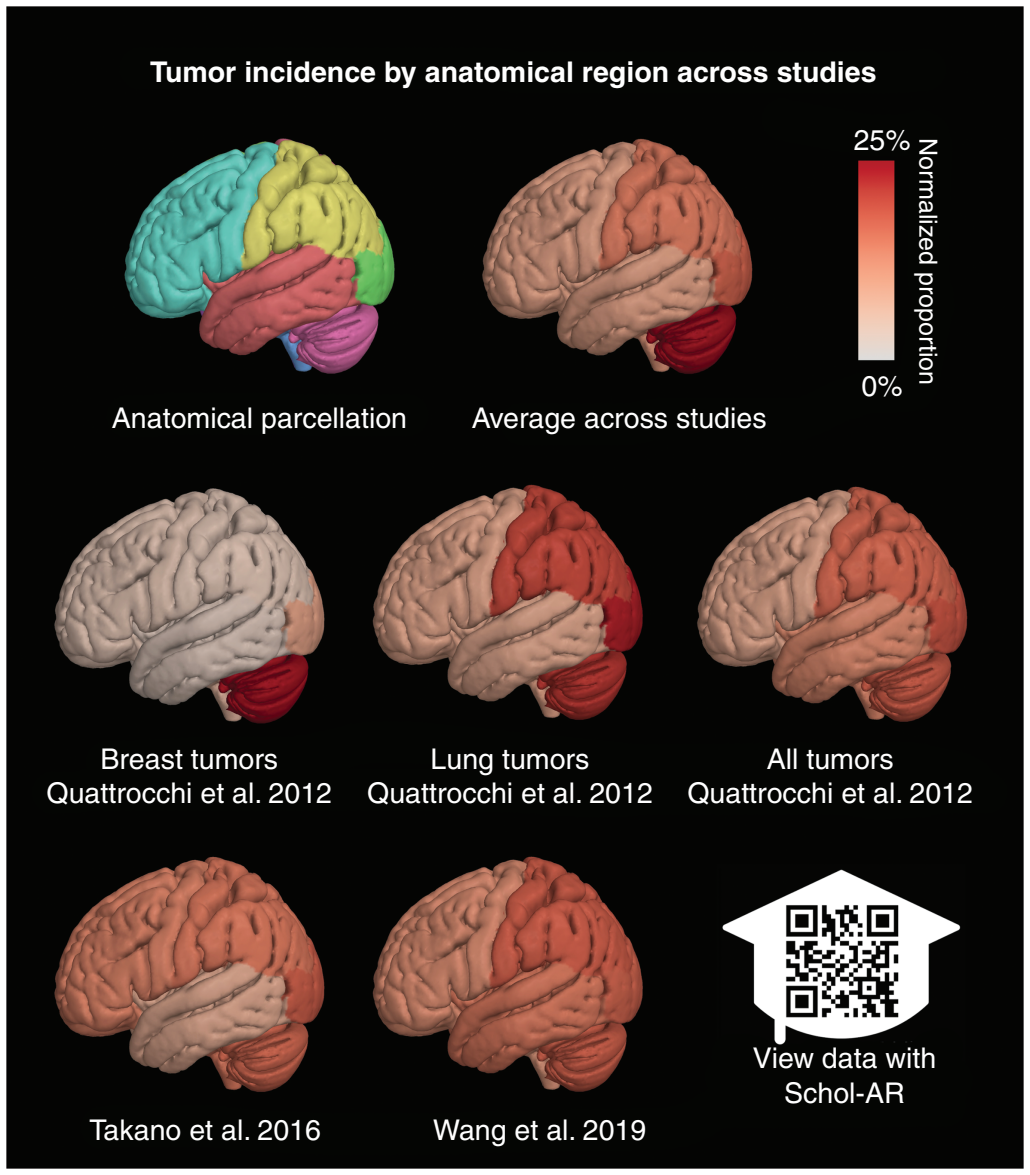
Surface renderings illustrating the patterns of anatomical localization of brain metastases reported in several papers included in the review. The images visualize probability estimates of tumor location assessed for each of the frontal, parietal, temporal, and occipital lobes, the brainstem, and the cerebellum (first row, first column). The surfaces are colored to show high probability in red (25% of cases including both hemispheres) and white (approaching zero probability). Probability estimates were computed by aggregating lesion extent from plots and tables of the listed reviews, normalizing by region volume, and normalizing. A composite plot was generated to show the average probability across all studies included in the figure. The data may be visualized in augmented reality using the mobile application Schol-AR, which can be found at https://www.schol-ar.io/. 3D visualizations were made using the Quantitative Imaging Toolkit (QIT) and with augmented reality functionality provided by Schol-AR (https://www.schol-ar.io/).^53^

Studies were subsequently analyzed for the association of primary cancer origin, molecular subtypes, and distribution of brain metastases. Some studies only reported the locations of metastases without analyzing their distribution or did not separate reported distribution based on pathology, and these were subsequently excluded from the analysis.^4,13–16^ The findings of each study were collected from reported data and compiled into summary tables. Inconsistent and heterogeneous reporting metrics between studies, as well as the emphasis on differing cancer types and patterns of anatomical distribution (eg, vascular, by lobes), precluded performing a meta-analysis. However, a summary figure was created based on the full data that were available in 3 studies^6,17,18^ (Figure 2).

## Results

A total of 13 articles met the criteria for inclusion. All studies were retrospective observational single- or multi-institution database reviews, thereby comprising Level IV evidence. In aggregate, these studies analyzed over 8227 brain metastases from 2599 patients. Eight reported the distribution of brain metastasis from breast cancers,^3,7,17,19–23^ including a total of 769 patients with more than 1352 brain metastases. Three did not list the number of metastases in patients in their study.^7,20,21^ The distribution of lung cancer brain metastasis was examined in 7 studies^3,6,17–19,22,23^ and included 1265 patients with over 5345 metastases. One study did not list the number of metastases analyzed.^6^ Brain metastases from melanoma, GI cancers, renal cell carcinoma, and prostate carcinoma were less well studied. Broken down by primary tumor type, these studies included over 611 patients with melanoma yielding more than 1761 brain metastases,^3,19,22–24^ more than 95 patients with over 341 brain metastases from GI primary cancer,^3,22–24^ over 106 patients with over 133 renal cell carcinoma brain metastases,^11,22,23^ and 103 patients with an unknown number of prostate carcinoma brain metastases.^25^

Studies used different methods to analyze the distribution of metastases. Two studies overlayed MRIs from all patients to create a single-image brain atlas and subsequently analyzed the metastases distribution based on voxels.^6,19^ Two studies enlisted a neuroradiologist to adjust each MRI to fit the Montréal Neurological Institute standard space atlas using the center of each metastasis as a guide.^3,21^ One study used a different standard template from subjects in the ICBM project with 2 radiologists creating lesion masks that accounted for the volume of the entire metastasis,^17^ and one study utilized stereotactic coordinates for the center of each metastasis from Gamma Knife treatment.^23^ One study mapped brain metastases onto cerebral vascular territory maps in the axial, coronal, and sagittal planes,^22^ and another compared the proportion of brain metastases in the cerebellum to the average proportion of cerebellar brain metastases.^24^ Five did not specify how they analyzed distribution.^7,11,18,20,25^ Findings are summarized below and in Figure 1, with additional details available in Tables 1–5.

**Table 1.** Studies Describing the Distribution of Brain Metastases From Lung Cancer

Study (year)	Type of Study (n)	Primary Cancer Subtype Analyzed (if available; n)	Distribution/Location of Brain Metastases
Bender et al. (2011)	Single-institution retrospective database review (85 patients, 227 metastases)	Non-small-cell lung cancer (n = 63 patients, 174 metastases), small-cell lung cancer (n = 22 patients, 53 metastases)	Lung cancer was more likely to metastasize to the cerebellum than what would be expected based on relative brain volume, with about 25% of metastases being found there.
Mampre et al. (2019)	Single-institution retrospective database review (195 patients, 316 metastases)	—	Metastases did not have a predilection for a particular vascular region.
Neman et al. (2021)	Single-institution retrospective database review (226 patients, 502 metastases)	—	Metastases were more likely to be in the left (0.67, 95% CI 0.47–0.82) and right temporal lobes (0.77, 95% CI 0.61–0.88).
Quattrocchi et al. (2012)	Single-institution retrospective database review (57 patients, 635 metastases)	Non-small-cell lung cancer (n = 51 patients, 611 metastases), small-cell lung cancer (n = 6 patients, 24 metastases)	Non-small-cell lung cancer metastases were more frequently found in the occipital lobes and cerebellum.
Schroeder et al. (2020)	Single-institution retrospective database review (167 patients, 1619 metastases)	Non-small-cell lung cancer (n = 120 patients), small-cell lung cancer (n = 46 patients), unknown (n = 1 patient)	Metastases were more likely to be in infratentorial areas.
Takano et al. (2016)	Single-institution retrospective database review (200 patients)	Adenocarcinoma (n = 129), small-cell carcinoma (n = 28), squamous cell carcinoma (n = 27), large cell carcinoma (n = 5)	Metastases were more likely to occur in the cerebellum, occipital, and temporal lobes (*P* = .012, *P* = .002, *P* < .001).
			EGFR L858R mutation metastases were more likely to occur in the caudate, cerebellum, and temporal lobes than those with exon 19 deletion (*P* = .02) and were located closer to the surface of the brain than those with exon 19 deletion (*P* = .0032) or wild-type EGFR (*P* < .0001).
Wang et al. (2019)	Single-institution retrospective database review (335 patients, 2046 metastases)	Adenomacarcinoma (n = 208), squamous cell carcinoma (n = 20), small-cell carcinoma (n = 74), large cell carcinoma (n = 2), other types (n = 13), unknown (n = 18)	Adenocarcinoma had the largest number of single metastases (39/71 patients, 55%; *P* < .05)
			Overall, the cerebellum (56%, 189/335 patients), right parietal lobe (54%, 182/335 patients), right frontal lobe (47%, 157/335 patients), and left frontal lobe (45%, 152/335 patients) had the highest rate of metastases, with no differences between these regions (*P* < .05).
			In patients with lung adenocarcinoma, the left (53%, 111/208) and right (48%, 100/208) frontal lobe and cerebellum (56%, 116/208) were more likely to have brain metastases (*P* < .05).
			In small-cell carcinoma brain metastases had a predilection for the cerebellum (61%, 45/74 patients) and right frontal lobe (46%, 34/74 patients; *P* < .05).
			In patients with squamous cell carcinoma, brain metastases were more likely to be found in the cerebellum (70%, 14/20 patients; *P* < .05).
			In patients with EGFR mutations, the left (62%, 23/37 patients) and right (62%, 23/37 patients) frontal lobes and cerebellum (57%, 21/37 patients; *P* = .998) had the highest rate of metastases, but it was insignificantly different than the distribution of wild-type EGFR metastases.

**Table 2.** Studies Describing the Distribution of Brain Metastases From Breast Cancer

Study (year)	Type of Study (n)	Primary Cancer Subtype Analyzed (if available; n)	Distribution/Location of Brain Metastases
Bender et al. (2011)	Single-institution retrospective database review (30 patients, 118 metastases)	—	Breast cancer was more likely to metastasize to the cerebellum than what would be expected based on relative brain volume.
Hengel et al. (2013)	Single-institution retrospective database review (57 patients)	—	HER2 expression was more common in cerebellar metastases, but it failed to reach significance.
Kyeong et al. (2017)	Single-institution retrospective database review (100 patients)	Triple-negative breast (n = 24), HER2+ (n = 48), ER+ or PR+ (n = 28)	No differences in number of brain metastases based on breast cancer subtype (triple-negative vs HER2+ vs ER+ or PR+).
			Top 10% regions for brain metastasis development are evenly distributed in triple-negative cancer, but had a predilection for the occipital and temporal lobe and cerebellum in HER2+ patients and for the frontal and occipital lobe and cerebellum for luminal subtype.
Laakmann et al. (2016)	Single-institution retrospective database review (300 patients)	ER+/− breast cancer (n = 141/120), PR+/− breast cancer (n = 125/135), HER2+/− breast cancer (n = 102/128), triple-negative (n = 51)	ER− patients had significantly more brain metastases than ER+ patients, and this same trend was seen for PR− and HER2− patients (*P* < .001).
			HER2+ patients had more cerebellar metastases than HER2− patients (*P* = .021).
			Patients with ER+ or PR+ tumor biologies had lower incidence of hippocampal metastases than those with ER− or PR−.
Mampre et al. (2019)	Single-institution retrospective database review (75 patients, 144 metastases)	—	Metastases were less likely to be located in PCA territory (n = 18, 13%; *P* = .01) and more likely to be in anterior–posterior watershed territories (n = 15, 10.4%; *P* = .05).
Neman et al. (2021)	Single-institution retrospective database review (134 patients, 285 metastases)	—	Metastases were most likely to be in the right cerebellar hemisphere (0.83, 95% CI 0.70–0.91).
Quattrocchi et al. (2012)	Single-institution retrospective database review (26 patients, 136 metastases)	—	Metastases were more likely to be located in the cerebellum.
Schroeder et al. (2020)	Single-institution retrospective database review (47 patients, 669 metastases)	—	Metastases favored areas of posterior circulation and were more likely to be in the cerebellum and less likely to be in the frontal lobes.

**Table 3.** Studies Describing the Distribution of Brain Metastases From Melanoma

Study (year)	Type of Study (n)	Distribution/Location of Brain Metastases
Bender et al. (2011)	Single-institution retrospective database review (29 patients, 73 metastases)	Melanoma was not more likely to metastasize to the cerebellum than the rest of the brain.
Mampre et al. (2019)	Single-institution retrospective database review (56 patients, 119 metastases)	Metastases from melanoma were more likely to be located in lateral lenticulostriate (n = 5, 4%; *P* = .03) and medial lenticulostriate (n = 2, 2%; *P* = .005) and less likely to be in cerebellar vascular territory (n = 11, 9%; *P* < .001).
Neman et al. (2021)	Single-institution retrospective database review (483 patients, 1099 metastases)	Metastases were most likely to be in the left temporal lobe (0.89 95% CI = 0.81–0.94).
Rogne et al. (2014)	Single-institution retrospective review (147 metastases)	Metastases were less likely to be in the cerebellum.
Schroeder et al. (2020)	Single-institution retrospective database review (43 patients, 323 metastases)	Metastases were more likely to be in the frontal lobes and avoid the cerebellum.

**Table 4.** Studies Describing the Distribution of Brain Metastases From GI Primary Cancers

Study (year)	Type of Study (n)	Distribution/Location of Brain Metastases
Mampre et al. (2019)	Single-institution retrospective database review (26 patients, 43 metastases)	Metastases from colon cancer did not have a predilection for a particular cerebral vascular territory.
Neman et al. (2021)	Single-institution retrospective database review (33 patients, 52 metastases)	Metastases from colon cancer were more likely to be in the right cerebellar hemisphere (0.45, 95% CI 0.26–0.65).
Rogne et al. (2014)	Single-institution retrospective review (71 metastases)	Metastases from colorectal cancers were more likely to be in the cerebellum.
Schroeder et al. (2020)	Single-institution retrospective database review (36 patients, 175 metastases)	Metastases favored the infratentorial space, with predilection for the cerebellum and avoidance of the frontal and parietal lobes.

**Table 5.** Studies Describing the Distribution of Brain Metastases From Renal Cell Carcinoma and Prostate Cancers

Study (year)	Type of Study (n)	Distribution/Location of Brain Metastases
*Renal cell carcinoma*		
Mampre et al. (2019)	Single-institution retrospective database review (44 metastases)	Metastases did not have a predilection for a particular cerebral vascular territory.
Neman et al. (2021)	Single-institution retrospective database review (89 patients, 168 metastases)	Metastases were most likely to be in the brainstem (0.47, 95% CI 0.27–0.68).
Seidel et al. (2015)	Multi-institution retrospective database review (17 patients)	Renal cell carcinoma metastases were more likely to be in deep white matter.
*Prostate*		
Tremont-Lukats et al. (2003)	Single-institution retrospective database review (103 patients)	Supratentorial-only brain metastases were found in 76% of patients and infratentorial-only in 21% of patients, with the remaining 3% having metastases in both compartments. No significant tendencies for metastases to travel to a particular location overall or by histologic subtype.

### Location and Distribution of Brain Metastasis From Lung Cancers

Lung cancer is the most common cancer to metastasize to the brain, consisting of 30–60% of all brain metastases, with 10–30% of patients developing brain metastasis.^26,27^ More specifically, the likelihood of developing brain metastasis in small-cell lung cancer (SCLC) is up to 40% within 1 year without radiation therapy, and clinical trials have demonstrated a survival benefit of prophylactic brain radiation.^26^ Non-small-cell lung cancer (NSCLC) has a lower incidence of brain metastasis, dependent on staging (18% of stage III cancers and 6–10% of stage I/II cancers without prophylactic brain radiation).^26^ Additionally, NSCLCs with exon 21 L858R point mutation of epidermal growth factor receptor (EGFR) have been reported to have more brain metastases than wild-type EGFR NSCLC.^6^ Our investigation of studies analyzing the distribution of brain metastases from all types of lung cancer found that they were more likely to occur in infratentorial areas, particularly the cerebellum.^3,6,17–19^ However, studies disagreed on other significant metastasis locations. One finding the parietal^18^ and frontal lobes^18^ were more common locations, and others observing the occipital^6,17^ and temporal^6,23^ lobes were more likely to contain a metastasis. These discrepancies may be explained by the subtypes of lung cancer included in each study.

The distribution of lung cancer brain metastases has been examined based on subtype (2 studies^17,18^) and genetic composition (2 studies^6,18^). Two found brain metastases from NSCLC arose more often in the occipital lobes and cerebellum.^17,18^ Within NSCLC, adenocarcinomas were most likely to travel to the frontal lobe (left—53%, 111/208 patients and right—48%, 100/208 patients). Brain metastases from squamous cell carcinoma were more likely to be found in the cerebellum (70%, 14/20 patients; *P* < .05).^18^ SCLCs tended to metastasize to the right frontal lobe (46%, 34/74 patients; *P* < .05) and cerebellum (61%, 45/74 patients; *P* < .05).^18^ Two studies looked at brain metastasis distribution based on the genetic profile. One found that brain metastases from primary lung cancers with EGFR L858R mutations were more likely to occur in the caudate, cerebellum, and temporal lobes than those with exon 19 deletions and were located closer to the surface of the cerebrum than those with exon 19 deletions or wild-type EGFR.^6^ On the other hand, Wang et al.^18^ found no differences in location based on mutation status, though did observe brain metastases occurred most in the left (62%, 23/37 patients) or right (62%, 23/37 patients) frontal lobes and cerebellum (57%, 21/37 patients) in patients with EGFR deletions (*P* = .998; Table 1).

### Location and Distribution of Brain Metastasis From Breast Cancers

Breast cancer is the second most frequent source of brain metastasis, and 10–30% of patients will develop brain metastases over the course of their disease.^26,28^ While younger age at diagnosis and higher tumor aggressivity (higher histologic grade and shorter time to first metastasis) are associated with a greater likelihood of developing brain metastasis in all breast cancer subtypes, differences in metastatic behavior do exist between breast cancer subtypes.^29^ Breast cancers expressing human epidermal growth factor receptor 2 (HER2) and triple-negative breast cancers are the most likely subtypes to metastasize to the brain, with brain metastasis occurring in 34% and 46% of patients, respectively.^29^ This is in contrast to luminal A (estrogen [ER] positive and/or progesterone [PR] positive/HER2-negative) breast cancers that metastasize to the brain in 14% of cases,^30^ and luminal B (ER-positive and/or PR-positive/HER2-positive) cancers that metastasize in 35% of cases.^31^ Laakmann et al.^7^ found when comparing tumors based on receptor expression that ER-negative patients had significantly fewer brain metastases than ER-positive patients (mean number of metastases: 15.26 vs 7.19, *P* < .001), with the same trend being true for PR (14.56 vs 6.95, *P* < .001) and HER2 patients (15.44 vs 8.24, *P* < .001). One study found no difference in mean number of brain metastases between triple-negative (5.33), HER2-positive (4.71), or tumors that expressed either ER or PR (5.35) (*P* = .88).^21^ However, this study was limited by smaller sample size (n = 100) and did find that triple-negative and HER2-positive cancers had a shorter mean time interval to onset of brain metastasis than other subtypes (triple-negative: 25.3 months, HER2-positive: 19 months, ER-positive or PR-positive: 42 months; *P* < .01).^21^

Topologically, the literature was generally consistent in reporting that breast cancer metastases from all subtypes were more commonly identified in the cerebellum and areas of posterior circulation.^3,17,19,23^ More specifically, Bender et al.^19^ found that 32/118 breast cancer brain metastases were located in the cerebellum, which was more than what was predicted based on relative brain volume. This was supported by Quattrocchi et al.^17^ and Schroeder et al.,^3^ though Quattrocchi et al.^17^ did not report on the number of metastases by location in their study. In addition to a predilection for the cerebellum (OR 2.16, *P* = .006), Schroeder et al. found breast cancer was less likely to metastasize to the frontal lobes (OR 0.49, *P* < .001).^3^ Studies examining distribution based on breast cancer subtype found HER2-positive breast cancers were primarily located in the cerebellum,^7,21^ occipital,^21^ and temporal^21^ lobes and less likely to be in the frontal lobe or subcortical region.^21^ In one study, the predilection of HER2-positive cancers for the cerebellum failed to reach significance.^20^ However, a separate study noted breast cancers that were ER-positive or PR-positive were less likely to be in the occipital lobe, subcortical region, or cerebellum than HER2-positive subtypes (*P* < .05).^21^ Additionally, ER-positive and PR-positive breast cancers were less likely than ER-negative or PR-negative subtypes to metastasize to the hippocampus.^7^ Triple-negative subtypes were more likely to be in the frontal lobe, limbic region, and parietal lobe when compared to other subtypes (*P* < .05) (Table 2).^21^

### Location and Distribution of Brain Metastasis From Melanoma

Melanoma has a high propensity for metastasis with an estimated 10–40% of patients developing brain metastasis.^32^ Predictors of metastasis to the brain in melanoma include skin ulceration, primary location on head and neck, and advanced tumor stage, particularly in unresectable disease.^32–35^ Studies analyzing the distribution of brain metastasis from melanoma generally found they were less likely to be in the cerebellum than other brain regions. Schroeder et al.^3^ found that 43 metastases from melanoma were more likely to be in the frontal lobes and less likely to be in the cerebellum. This was supported by Bender et al. who found 73 melanoma metastases were less likely to be located in the cerebellum than in another part of the brain,^19^ Mampre et al.^22^ reported only 11 out of 119 brain metastasis from melanoma were in the cerebellum (*P* < .001), and Rogne et al.^24^ and Neman et al.^23^ found brain metastasis from melanoma were most likely to be found in the left temporal lobe. These studies suggest melanoma may be more likely to metastasize to different brain regions than breast and lung cancers (Table 3).

### Location and Distribution of Brain Metastasis From GI Cancers

GI cancers have a much lower incidence of brain metastasis, with less than 1% of pancreatic and gastric cancers and up to 4% of esophageal and colorectal cancers metastasizing to the brain.^36^ Out of 4 studies statistically analyzing the distribution of brain metastases from GI cancers, 3 included metastases from colorectal primary cancers^22–24^ and 1 lumped together metastases from a variety of GI primaries.^3^ These studies overall found metastases were more likely to be in the cerebellum and less likely in the frontal and parietal lobes.^3,23,24^ The study analyzing distribution by vascular territory did not find a significant predilection for any area, though had a small sample size (43 metastases; Table 4).^22^

### Location and Distribution of Brain Metastasis From Renal and Prostate Cancers

Renal cell and prostate carcinoma metastasize to the brain in 2–16% and less than 1% of cases, respectively.^37,38^ Predictive factors for these cancers to metastasize to the brain are less well studied, though in prostate carcinomas it has been seen that there may be differences in the likelihood of brain metastasis based on subtype.^25^ Tremont-Lukats et al. found no significant tendencies for brain metastases from prostate carcinoma to travel to a particular location overall or by histologic subtype in a study of 103 patients. The authors did note that supratentorial-only brain metastases were found in 76% of patients and infratentorial-only in 21% of patients, with the remaining 3% having metastases in both compartments.^25^ Renal cell carcinomas appeared to be more likely to metastasize to deep white matter regions, including the brainstem, though when analyzed by vascular territory there was no significant association for a particular area (Table 5).^11,22,23^

## Discussion and Future Considerations

This systematic review of contemporary literature investigating the topographical patterns of distribution of brain metastases based on primary systemic cancer found general agreements in the locations of brain metastases from breast, lung, and melanoma cancers. However, there was significantly less data on the distribution tendencies of breast and lung cancer subtypes as well as all types of GI, prostate, and renal cancers. Specifically, breast cancer demonstrated a consistent pattern of metastases to the cerebellum and areas of posterior circulation, with differences in number and distribution based on subtype. Studies investigating lung cancer brain metastases demonstrate a similar pattern distribution seen in breast cancer, with the cerebellum being a primary area of metastasis and a predilection for the posterior fossa. Melanoma tended to metastasize to the frontal and temporal lobes, though it was only investigated in 3 studies. It appeared that colorectal cancers followed similar patterns of metastasis to breast and lung cancers, with brain metastases primarily being found in the cerebellum, though data are generally limited. The dearth of studies statistically analyzing the distribution of metastases from renal and prostate cancers and the mixed GI pathologies included in one study prevent conclusions from being drawn about their metastasis patterns. Improved investigation into these patterns, particularly based on cancer subtype and genetic makeup, will be important in determining the significance of topological patterns. The authors hypothesize that the differential spatial topographic patterns of metastases arising from various primary cancers and molecular subtypes identified in this review provide insight into a yet undiscovered relationship between primary cancers attempting to metastasize to various brain regions and the local tumor microenvironment. The facilitatory and inhibitory signals that underlie this dynamic process, including potential paracrine signals, neurotransmitters, and/or neuronal signals may one day be harnessed to prevent or curb the process of cerebral metastasis.

There are numerous hypotheses for why brain metastases have certain predilections for a particular region of the brain. The “seed and soil” hypothesis, where the tumor (“seed”) metastasizes to a specific area in the brain (“soil”) due to its unique microenvironment that attracts and allows the tumor to grow, was initially postulated by Paget.^8^ Arterial hematogenous spread is the primary method by which tumors metastasize to the brain. Studies have demonstrated the critical role the neurovascular endothelial cells and their protein expression profile likely play in this process.^39,40^ More precisely, it has been shown that cellular communication between the tumor cells, brain pericytes, astrocytes, and vascular endothelial cells are at least in part responsible for the growth and proliferation of the vasculature required for tumor cell progression.^41,42^ Theories for the propensity of metastases to travel to the cerebellum, in particular, include its higher gyral density (and therefore increased cortico-junctional surface) compared to that of the cerebral hemispheres,^17^ higher blood volumes and longer perfusion times in areas of posterior circulation,^43,44^ and varying regional vasomotor responses to signaling or unique innervations in the parieto-occipital and cerebellar regions that favor a state of greater vessel dilation.^45–48^

In addition to anatomic considerations, the primary tumor likely influences both the “seed” and the “soil.” Melanoma, NSCLC, and breast cancer have been shown to co-opt vasculature and grow along existing vessels, while other lung cancers instigate early angiogenesis to drive perivascular growth.^9–11^ These effects are caused by different cellular receptor and protein expressions, unique to each tumor type. In breast cancer, various chemokine receptors have been identified that affect the propensity of a tumor to metastasize to the brain and its location, including CXCR4, CCR7, COX2, and EGFR.^21^ While initially only seen in animal studies, human studies comparing the genetics of primary tumors and their corresponding brain metastases have proved the existence of mutations present only in brain metastases, indicating branched evolution.^49^ For example, there is known discordance between the molecular subtype of primary breast cancer and corresponding brain metastases in up to 20% of cases, usually consisting of loss of ER/PR and gain of HER2 expression.^30^ All of the studies in this review analyzed patterns based on immunohistochemistry of primary breast cancer, indicating further investigation is merited to determine the role molecular changes play in metastasis.

In lung cancer, there were differences in the distribution of adenocarcinoma, squamous cell carcinoma, and SCLC, which may result from distinct biological behaviors. For instance, adenocarcinoma is known to infiltrate the bronchi and spread hematogenously and SCLC exhibit rapid growth and malignant behavior, while squamous cell carcinomas only invade blood vessels in advanced disease states.^18^ Our review additionally found different EGFR mutations modified the predilection of a tumor to metastasize to a particular brain region. It has been previously shown that lung cancer patients with EGFR mutations have more brain metastasis than those with wild-type EGFR, with the incidence dependent on the type of EGFR mutation.^18^ One confounding factor when comparing mutational status is that genomic assays used by different studies vary in their sensitivity, methodology, and reportable range. For this reason, variability in the analytical assays used by research groups may account for some failure to reproduce findings in brain metastasis distribution based on EGFR mutations or deletions. Nonetheless, the reported findings reiterate the significant role a tumor’s genetic profile plays in its brain tropism and underscore the potential for targeted therapeutics specifically for brain metastases.

One of the main challenges in analyzing the distribution of brain metastases is the difficulty in modeling the shape and structures of the brain. Studies in this review used various techniques to investigate distribution patterns, with some utilizing the center of the metastases as a guide and others analyzing based on metastasis volume. Brain metastases are generally round in shape and develop concentrically, so for modeling purposes, the exact center of the metastases is often used to determine its location.^6^ Different MRI resolutions and thickness of imaging cuts are liable to affect spatial modeling, and modeling differences likely account for much of the discrepancies noted between studies in this review. Newer and more advanced modeling techniques would allow for a better understanding of brain metastasis patterns.

Continuing research aimed at understanding the distribution of brain metastases based on primary cancer is essential for multiple reasons. In some cases, brain metastases are discovered before the primary tumor is identified. Providing clinicians with a list of likely places to start examining would be beneficial, particularly in the absence of other telling clinical signs or symptoms.^50^ Additionally, no primary tumor source is identified despite imaging workup in up to 15% of patients.^51^ Perhaps more importantly, as WBRT is a primary treatment method for brain metastases, shrinking the radiation field to the tumor-specific distribution of metastases would improve therapeutic outcomes and minimizing unwanted consequences of radiation therapy.^3^ Finally, it is essential to note that autopsy studies demonstrate a higher incidence of brain metastases than what is reported on neuroimaging, likely due to clinically silent or undetectable metastases.^40^ The locations of these brain metastases would be central to understanding distribution of metastases based on primary cancer.

### Limitations

All the studies in this review were retrospective in nature, thereby limiting the strength of the analyses and available level of evidence. Database studies are limited by coding errors and omissions by non-physician coding staff, missing data, and dependence on the accuracy of available coding categories.^52^ The heterogeneity in data reporting of the source studies makes this review a review of the available literature which likely differs slightly from real-world data. While we felt it was important to include all published literature utilizing MRI to analyze brain metastasis distribution, there are limitations associated with including studies over such a broad time period. The heterogeneity in studies reporting distribution of brain metastases based on genetic subtype or lumping all subtypes of one cancer together makes it difficult to draw conclusions. Additionally, brain imaging was historically utilized only for new neurologic symptoms rather than as a screening tool, which may influence the propensity of metastases for the cerebellum where new metastases more rapidly cause symptoms. Many of the studies were somewhat contradictory, likely due to varying methods of distribution analysis, interpretation, and tumor characteristics not specifically investigated or captured by available data, as well as differences in the number of patients/metastases available for analysis. In addition, different treatments may influence metastatic patterns and therefore brain metastasis locations. Finally, understanding the distribution of brain metastases based on primary cancer is limited by the ability of a research team to characterize the primary lesion, which is not possible in up to 15% of patients.^51^ Lack of data reporting and differences in reporting metrics precluded our ability to perform a meta-analysis and increase the significance of our findings.

## Conclusion

This systematic review investigating current literature on the distribution of brain metastases demonstrates key patterns of metastasis, based both on the primary cancer type and molecular subtype. While there is likely a nuanced distribution based on genetic subtype, we found that brain metastases from breast and lung cancer generally were more frequently located in the occipital lobe and cerebellum, while melanomas had a predilection to metastasize to the frontal lobe. This review also underscores the need for further research in this field, with inconclusive results regarding the distribution patterns of prostate and renal cancers. Potential differences in tumor microenvironment, together with intrinsic tumor factors, likely play important roles in determining its predilection for a particular brain region. Future studies should be focused on analyzing data from many metastases and patients and considering tumor and patient characteristics to better understand how these factors influence the distribution of brain metastasis and the potential therapeutic significance.
